# Procuct news

**DOI:** 10.1155/S1463924682000388

**Published:** 1982


Journal of Automatic Chemistry, Volume 4, Number 3 (July–September 1982), pages 147–156

### Product news

*A part of this issue's new products section is devoted to instruments launched at the recent Pittsburgh conference. The meeting was held from 8 to 13 March 1982 at the Atlantic City Convention Hall, in New Jersey, USA. Next year's Pittsburgh conference on analytical chemistry and applied spectroscopy will also be at Atlantic City and will run from 7 to 12 March.*

### Programmable wavelength detector—UV-100

Varian announced a fully integrated programmable wavelength detector at Pittsburgh. The detector provides automated UV detection on the company's Model 5000 series of liquid chromatographs.

Chromatographers may select any number of wavelengths in 1 nm increments between 190 nm and 370 nm for optimal detection of all components in a run. Simple keyboard entries control range, baseline setting and time constant; these automated functions may be changed at any point during operation and as often as desired.

To improve data accuracy and report quality, peaks can be attenuated and the baseline may be reset without disrupting operations. The time constant, which is program specific, may be set at 0·05, 0·5, or 5·0 s.

The UV-100 fits inside the Model 5000 liquid chromatograph, so no additional bench space is required. The UV-100 flow cell and its optics are combined in a unique self-centring assembly designed to maintain perfect alignment. Hemispherical lenses at each end of the flow cell define a cylindrical illuminated volume between slit and grating images. This design achieves maximum light throughput with minimum wall-induced refraction index effects.

For trace analysis studies, a stable, low-noise baseline allows the UV-100 to provide high sensitivity detection. Under normal operating conditions, peak-to-peak baseline noise is $5 \times 10^{-5}$ AU. Another notable feature is the linearity of $\pm 1\%$ to AU, which permits users to quantitate reliably over a wide concentration range with one calibration standard.

The UV-100's small flow cell, fast time constant and closely coupled design also provide excellent performance for both normal and ultra-fast LC applications.

The UV-100 is available both as a factory-installed option and as a field upgrade for the 5000 Series and VISTA Liquid Chromotographs. The result is enhanced throughput and lower cost per analysis for Varian LC owners.

*Further information from Jack Bell, Varian Instrument Group, 2700 Mitchell Drive, Walnut Creek, California 94598, USA.*

**Circle No. 6 on Reader Enquiry Card**

### XL-300 superconducting FT NMR spectrometer

The XL-300 is a 300 MHz superconducting Fourier transform nuclear magnetic resonance spectrometer. It is intended for research applications and, apparently, it surpasses the sensitivity of most high field systems and is 50% cheaper.

Advances in probe technology have produced a guaranteed 300-to-1 signal-to-noise (S/N) ratio using a 10 mm probe and an ASTM sample. This is the highest ASTM sensitivity specification in the industry. For the user, this superior sensitivity reduces the minimum sample size required for meaningful results and allows successful experiments to be run on short-lived or unstable samples. Dispersion characteristics are such that spectral interpretation is easier, allowing the solution of problems usually considered possible only on more costly instruments with much higher field strengths.

The software is based on PASCAL and provides message displays, parameter calculations, branch points and many other operations automatically.

The XL-300's correlated 2-D capabilities produce faster results on complicated experiments. Answers that require hours of multiple runs or multiple experiments on other NMR systems are possible on the XL-300 in only a fraction of that time.

A two-level instrument control system consisting of two independent processors with separate memories maximizes flexibility. One performs data manipulation and experiment management; the other monitors data axquisition and provides automatic instrument control. The result is that a single XL-300 operator can transform, plot, display and acquire in as many as nine different experiments concurrently.

*Further information from Bob Sheldon, Varian Instrument Group, 611 Hansen Way, Palo Alto, California 94303, USA.*

**Circle No. 7 on Reader Enquiry Card**

### Surface area and pore structure analysis

Micromeritics introduced a completely automatic surface area and pore volume analyser—the DigiSorb 2600—at Pittsburgh. The instrument measures surface area and pore structure of a wide variety of industrial materials including catalysts, carbons, ceramics, clays, metal oxides, ores and fuels. DigiSorb 2600 automatically determines adsorption and/or desorption isotherms, it calculates the B.E.T. surface area, and determines the distribution of pore volume and pore area versus pore diameter. Data are generated in both tabular and graphical form. Up to five samples can be analysed automatically without reloading. Instrument operation and data acquisition, reduction and print-out are all microprocessor-controlled. Communication is by a teleprinter, and an auxiliary RS-232 serial interface is available. Programming is operator-orientated in Fortran IV on a 5·25 in. floppy disc.

The analyser provides versatile, standardized analysis routines: sample parameters, modes of operation etc., can easily be selected and modified to suit different analyses. Up to 150 relative pressure points can be used to measure the adsorption and/or desorption isotherm for each sample. Other user features of the instrument are an alphanumeric display for prompting messages, and an operation status display for up-to-the-minute monitoring of analysis.

*For more information contact Micromeritics Instrument Corporation, 5680 Goshen Springs Road, Norcross, Georgia 30093, USA.*

**Circle No. 8 on Reader Enquiry Card**

### Active catalyst surface analyses

Micromeritics also launched their ChemiSorb 2800 at the Pittsburgh meeting. The machine provides automated sample preparation and chemical adsorption to determine catalyst activity. Active surface in a catalyst is measured by exposing a prepared sample to a reactive gas at a controlled temperature. The ChemiSorb can also be used to measure any gas uptake by solids at temperatures from ambient to 750°C (for example for carbon dioxide on chars in synfuels). Up to five samples are prepared and analysed automatically, a teleprinter is supplied and an auxilliary RS-232 serial interface is available. The machine is programmed in Fortran IV.

Four basic chemisorption routines are provided to meet most analysis requirements: two for hydrogen and one each for oxygen and carbon monoxide. Sample parameters and modes of operation are easily selected and can be modified to suit different analyses.

Precise temperature control permits reproducible results and prevents destructive transformations to certain catalysts; and pressure, from a few $\mu$m Hg to over 900 mm Hg, can be equilibrated with each sample. A flow-through preparation system speeds sample clean-up, particularly when dealing with dirty catalysts removed from process.

*More information on the instrument can be obtained from the Micromeritics Instrument Corporation.*

**Circle No. 9 on Reader Enquiry Card**

### The Videochart Recorder

The VideoChart Recorder is being promoted by its developer, Mr H. G. Matthews, as providing an ideal solution 'to the problem of converting an analytical laboratory into a productive, computer-oriented facility in an economical, controlled and people-sensitive manner'. The VideoChart Recorder is a user-interactive data acquisition, manipulation and display instrument which combines the functions of a chart recorder, integrator and scientific calculator with video presentation and an RS232C computer interface. The Recorder does not need any software, it easily connects to laboratory instruments and computers and it presents data by combining graphics with numerical values under real-time control.

The Recorder was demonstrated at Pittsburgh connected to an Apple microcomputer. Conferees were shown how, for an investment of around $8000, a combination of the instrument and a micro could be used in laboratories which do not have a full-scale computer system. The 'personal' computer stores and retrieves data records by ID number using floppy discs or cassettes.

*Details from H. G. Matthews, Instrumentation Graphics, 60 Church Street, Yalesville, Connecticut 06492, USA.*

**Circle No. 10 on Reader Enquiry Card**

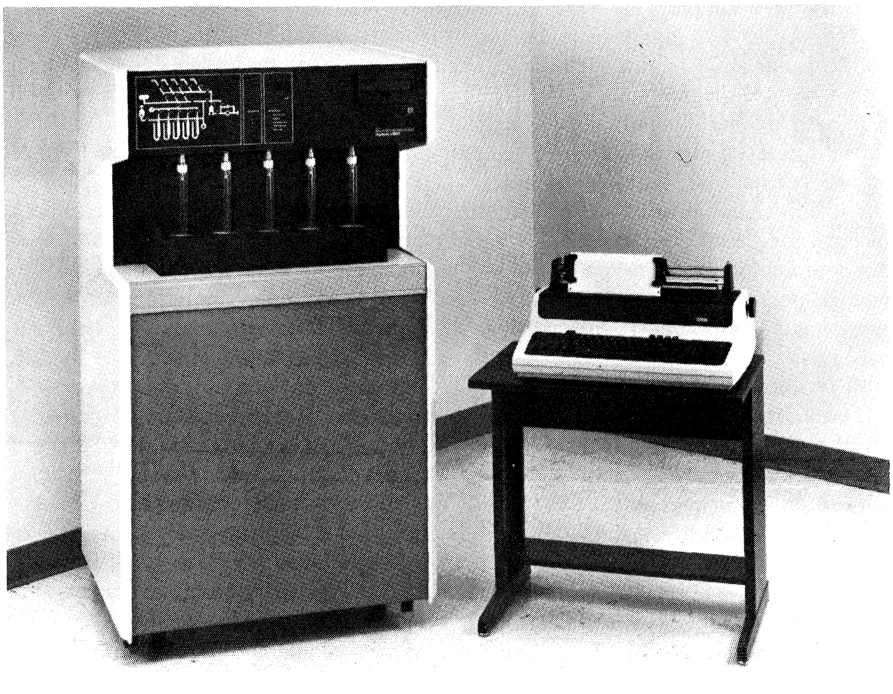

*The ChemiSorb 2800 launched by the Micromeritics Instrument Corporation at Pittsburgh.*

### New HPLC series from Kontron Analytical

A new family of high-pressure liquid chromatography (HPLC) modules, which offer automatic injection and processing of 120 samples, were launched by Kontron Analytical at the Pittsburgh Exposition. Called the HPCL Series 600, the instruments reduce the most time-consuming step in HPLC: the complete preparation of a sample prior to analysis. The series also features automatic method development, in which one can determine which column and mobile phase are necessary for the separation.

The main components of the Series 600 are:

*The MSI 660 autosampler* which provides reproducible injections of up to 120 samples using minimal sample volumes. Injections are controlled by a microprocessor via a pneumatically driven injection-valve followed by a rinse and dry cycle of the entire vent cross-contamination between samples. When the sampler is used with the Kontron Model 200 programmer the analysis time and the repetitions per sample can be altered as required. The sample volume can vary between 30 $\mu$l and 4 ml, while the injection volume ranges from 10 $\mu$l to 2 ml.

*The LCS 610 isocratic system.* The system has a digitally controlled single piston pump and a constant flow-rate that can be digitally set between 0·1 and 9·9 ml/min., or up to 28 ml/min. with a preparative pump head. Up to four standard columns can be installed in the

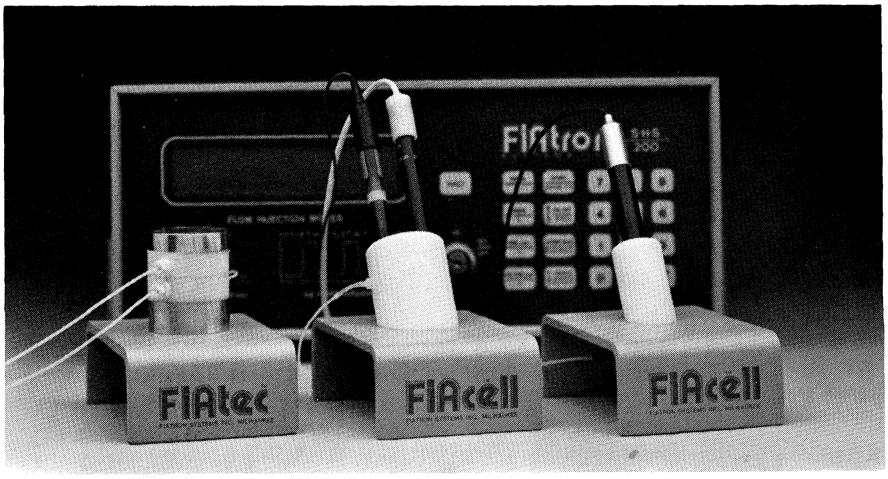

*The microprocessor-controlled solution handling system from FiAtron. The FiAcells and the FiAtec are used with the system to automate pH, redox and ISE measurements.*

column chamber which may be thermostatically controlled if required. The system is compact but the pump head, connections and valves can be easily checked and changed.

*The LCS 620 gradient system*, which is designed for methods development in research laboratories. The single piston pumps along with a small volume dynamic mixer, allowing complex gradient profiles to be used for difficult separations.

*The LCS 630 isocratic system with autosampler*—the 630 is a self-contained system controlled by a micro. Number of samples, number of analyses per sample and analysis time are entered via a keyboard; the 630 is intended for routine laboratory use.

*LCS 640 gradient system with sampler*, built for research and quality-control laboratories. The microprocessor controlled autosampler, in conjunction with the Model 200 programmer, provides random access of samples allowing automatic method development of even the most difficult separations and processing of different samples. Combination with the eluent switching system, LCS 870, allows the further optimization of routine analyses or multidimensional separations.

*The MCS 670 tracer*. Any type of enrichment on guard columns is possible with the tracer. Combinations of various switching methods and column materials allow even the most complex samples to be easily and reproducibly analysed. Up to 12 different eluents can be used.

*Prices for each HPLC Series 600 configuration will be quoted individually, more information from Kenneth Moilanen, Kontron Analytical, 630 Price Avenue, Redwood City, California 94063, USA.*

**Circle No. 11 on Reader Enquiry Card**

### FiAtron's SHS-200

An advanced microprocessor-controlled flow-injection instrument, the SHS-200, was displayed at Atlantic City by its manufacturer: FiAtron. The SHS (Solution Handling System)-200 is designed to significantly increase the analytical productivity of any lab—for both high- and low-volume work-loads. The machine automates most solution-handling tasks and is readily interfaced with virtually any analytical flow-through instrument. The SHS-200 controls a dedicated multichannel peristaltic pump, a dual-channel sample injector and an optional sample changer. All components are under software control and are readily programmable from the soft-touch front panel keyboard. For example, the solution flow rate (typically 3·0 ml/min.) can be readily changed without resorting to time-consuming tube changes by simply reprogramming the pump motor speed. The injected sample volume (2 μl minimum) can also be readily re-programmed without any sample loop changes.

In addition to the 'transient' signals usually found in flow-injection analysis, the SHS-200 can also generate 'steady-state' signals of any width, by programming the injection of larger sample volumes. Typically, sample injections greater than 200 μl give rise to 'steady state' signals.

Reagent addition can be carried out on a continuous or an intermittent basis by means of a programmable reagent value. For efficient mixing, the reagent is injected into the carrier stream via a Tangental Flow Chamber (volume 4 μl) which creates a localized flow region of high turbulence. A fourth pumping channel allows additional, continuous reagent injection. A total of three different reagent additions can be accommodated by the SHS-200.

In the 'Stop-Flow' mode, slow chemical reactions (for example enzyme reactions requiring incubation) can be readily accommodated. The SHS-200 pumps a programmable volume of sample and reagent into the detector. The sample-reagent mixture remains stationary in the detector for a programmable time interval during which measurements can be carried out.

When calibrated, the SHS-200 can be used as a solution dispensing device. A programmable volume of solution can be metered into the containers of a sample changer. The automated, repetitive dispensing of precise volumes of solutions into large arrays of test-tubes is possible in this mode.

Applications include AA, AE, ICP and flame photometry; liquid chromatography; UV/Visible, IR, fluorescence.

*Further information from Customer Service, FiAtron Systems Inc., PO Box 17927, Milwaukee, Wisconsin 53217, USA.*

**Circle No. 12 on Reader Enquiry Card**

### Sentinel—an automated liquid chromatograph

Sentinel is the first liquid chromatograph that can optimize separations in 12–24 h using four solvents and the seven experiment technique. The chromatograph is the latest addition to Du Pont's series 8800 HPLC system—it is part of the 8800's modular design and can be added to any of the instruments in the series. With Sentinel, chromatographers can free themselves from hour-to-hour operation during methods development—operators simply enter column number, a desired k' or retentivity value, and the number of sample vials to be used: the machine then proceeds automatically.

The system has five steps called 'Sample', 'Scout', 'Search', 'Separate' and 'Sort'. They are based on the solvent triangle concept developed by L. L. Snyder and a Du Pont chromatographic routine termed 'selectivity optimization'. The triangle concept reduces solvent/sample interactions to three types: proton acceptor, proton donor and dipole-dipole. A predictive model uses the concept to optimize strength and selectivity using four solvents for improved separations in reversed phase liquid chromatography.

Sentinel automates the triangle concept and the optimization technique, reducing them to routine practice.

'Sample' is a basic, initial phase of Sentinel that provides automatic control of the overall sampling sequence required to execute the total program. Prior to this step, the analyst has provided the system with column void volume, a desired k' or retentivity value, and the number of standards to be used. The instrument provides auto-injection and autosampler functions, as it adjusts time sequences to fit the experiments. Based on information entered by the chromatographer, 'Scout' then runs a routine standard gradient to determine if the LC mode selected and isocratic method are feasible. Desired retention time and column volume are next used to predict mobile phase concentration, using the first of three isocratic binary mobile phases (usually methanol/water) needed to produce the desired retention. 'Search' sets up the experimental conditions predicted by 'Scout', runs the predicted mobile phase concentration and compares the results with the predicted value. If the values differ, it will re-predict the mobile phase and continue this procedure until the chromatographic data match the analyst's retention requirement. 'Search' calculates compositions of the mobile phase for the other two points of the solvent/selectivity triangle, such as acetonitrile and THF, and runs chromatograms using these predictions until it obtains acceptable retention results. The fourth step, 'Separate', uses 'Search' results to automatically run all of the seven experiments that are part of the technique to optimize solvent strengths. The data from this step is then organized and reported by 'Sort'. 'Sort' is used when chromatographic standards are incorporated into the methods development routine. It numbers the peaks in the seven experiments, and recognizes and highlights in printed format all changes in elution order.

Each step can run automatically or upon instructions from the operator.

*Further information from the Du Pont Company, Analytical Instruments Division, Concord Plaza, McKean Building, Wilmington, Delaware 19898, USA.*

**Circle No. 13 on Reader Enquiry Card**

### 'Techscan'

The March 1982 of Du Pont's newsletter, *Techscan*, contains abstracts of technical papers presented at Pittsburgh. It also has two feature articles: 'Four-solvent HPLC: state of the art', and 'Interferon characterization via HPLC'; a description of 'Safeguard', a new column plan developed by Du Pont, and a presentation on HPLC of proteins and peptides by German researchers.

*'Techscan' is available from the Wilmington branch of Du Pont (as above).*

**Circle No. 14 on Reader Enquiry Card**

### Viscometer for consistency control

The consistency of many types of products can be determined with Nametre's new Model 7·010 viscometer. Each determination tests consistency as quickly as a control technician can test with his fingers. The machine's quantitative viscosity display is accurate to $\pm 2\%$ and it is reproducible to $\pm 0{\cdot}1\%$ over the range 0·1 to 100 000 centipoise. The small stainless-steel tip can be used to sense the viscosity of adhesives, blood, grease, honey, ink, jelly, ointment, sea-water, soap and toothpaste for example. The temperature of the sample is also displayed.

*This instrument is available from the Nametre Company, 1778 State Highway 27, Edison, New Jersey 08817, USA.*

**Circle No. 15 on Reader Enquiry Card**

### LABNET

Spectra-Physics chose Pittsburgh as the launch pad of their 'Expandable Chromatography Communication System': LABNET. The purpose of LABNET is to provide the maximum degree of flexibility, expandability and reliability possible in Spectra-Physics' microprocessor-based instruments at the lowest possible cost. LABNET is made up of a number of functions: a signal

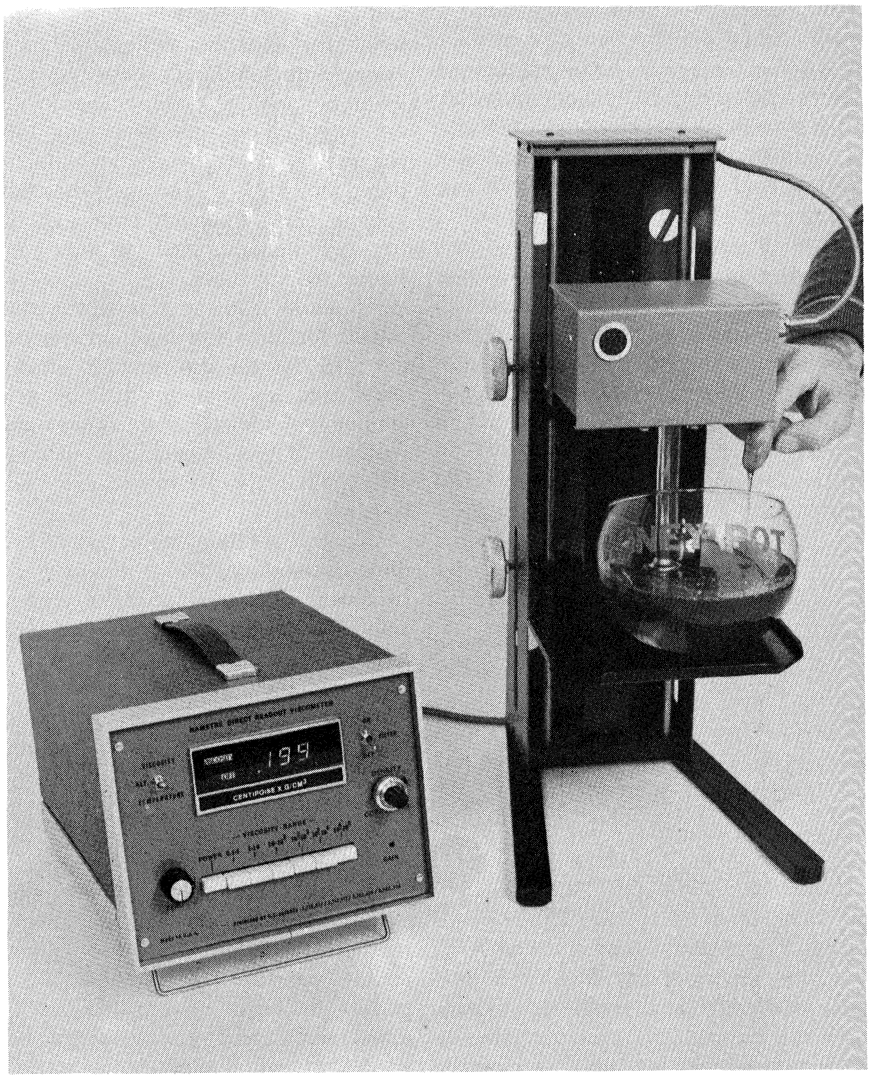

*The Model 7·010 viscometer for testing product consistency. (Nametre Corporation, Edison, USA.)*

pathway, a vocabulary, a grammar, a syntax and rules of order. The system can easily be integrated with four of the company's instruments: the SP8100 automated HPLC, the SP7100 high-performance GC, the SP4200 computing integrator, the SP8700 solvent delivery system—these already have the hardware, intelligence and command structure to provide the communication link.

*The use of LABNET is described in an article by M. P. T. Bradley in the February 1982 edition of Spectra-Physics' 'Chromatography Review', which is available from Spectra-Physics, Autolab Division, 3333 N. First Street, San Jose, California 95134, USA.*

**Circle No. 16 on Reader Enquiry Card**

### TAN/TBN—a new titration system

A new titration system was introduced by Fisher Scientific at Pittsburgh. The TAN/TBN determines total acid and total base numbers in oils. It is fully automated, does not need a skilled operator and the system is quick enough to be used for routine maintenance. TAN/TBN's analytical method is derived from the industry standard—ASTM D664.

The heart of the system is the Titrimeter II FEP Titrator, which automatically titrates a single sample to one or two fixed endpoints, pre-set via digital switches. Operating over a $-1000$ to $+1000$ mV or 0 to 14 pH range, it automatically delivers titrant with an accuracy of $\pm 0{\cdot}1\%$ of syringe volume and $\pm 0{\cdot}02\%$ precision, and proportionally approaches end-points so that titrant delivery rate continually decreases as the endpoint is approached.

The TAN/TBN includes all of the peripheral equipment, supplies, and reagents needed to begin operation.

The operating instructions are pictorially presented and designed to be easily used by an inexperienced operator.

*More information from Fisher Scientific Company, 711 Forbes Avenue, Pittsburgh, Pennsylvania 15219, USA.*

**Circle No. 17 on Reader Enquiry Card**

### Autosampler and ICAP spectrometer

The Jarrell-Ash Division of the Fisher Scientific Company displayed several products at Pittsburgh including an autosampler accessory for their ICAP-9000 spectrometer and the AtomScan 2000.

The autosampler accessory is computer controlled and complements the ICAP-9000 simultaneous spectrometer. Featuring a maximum capacity of 114 samples, the autosampler processes large sample work-loads at a rate of one sample/min. The machine fits easily onto a lab cart or work bench. The Jarrell-Ash autosampler is linked directly to the ICAP-9000 spectrometer by an intelligent processor/controller. This enables the user to completely program the autosampler for rinse times, sample flush times, and exposure times. It automates several operating functions of the ICAP-9000 spectrometer: instrument standardization, quality-control monitoring, and concentration limit monitoring. The autosampler features a continuous flow rinse station, manual reset push-button, and built-in drip tray for spill protection.

Jarrell-Ash's AtomScan 2000 is a sequential inductively coupled argon plasma (ICAP) spectrometer for methods development and single- or multi-element analyses. The AtomScan 2000 has a high-speed drive which can traverse the entire UV-VIS spectrum in less than 1 s. This feature is a significant advance over existing stepper-motor technology. The AtomScan 2000 scanning monochromator has a 0·75 m crossed Czerny-Turner optical design which covers a wavelength range from 175 to 800 nm. To assure high wavelength accuracy and repeatability, the AtomScan 2000 is microprocessor-calibrated against known wavelengths at frequent intervals during use. Additionally, the instrument is temperature-controlled and air-cushioned for stability.

To analyse a variety of solution samples, the AtomScan 2000 uses an ICAP source powered by a 2500 W, 27 MHz RF generator. The analyst uses the AtomScan data-acquisition system—computer, video monitor and printer—to operate the instrument. Mini-floppy discs contain AtomScan software programs; and menu-driven software leads the analyst step-by-step through instrument operation. A high resolution graphics display format is included for easy interpretation of spectral scans.

*For more information contact: Jarrell-Ash Division, Fisher Scientific Company, Evanthia Malliris, 590 Lincoln Street, Waltham, Massachusetts 02254, USA.*

**Circle No. 18 on Reader Enquiry Card**

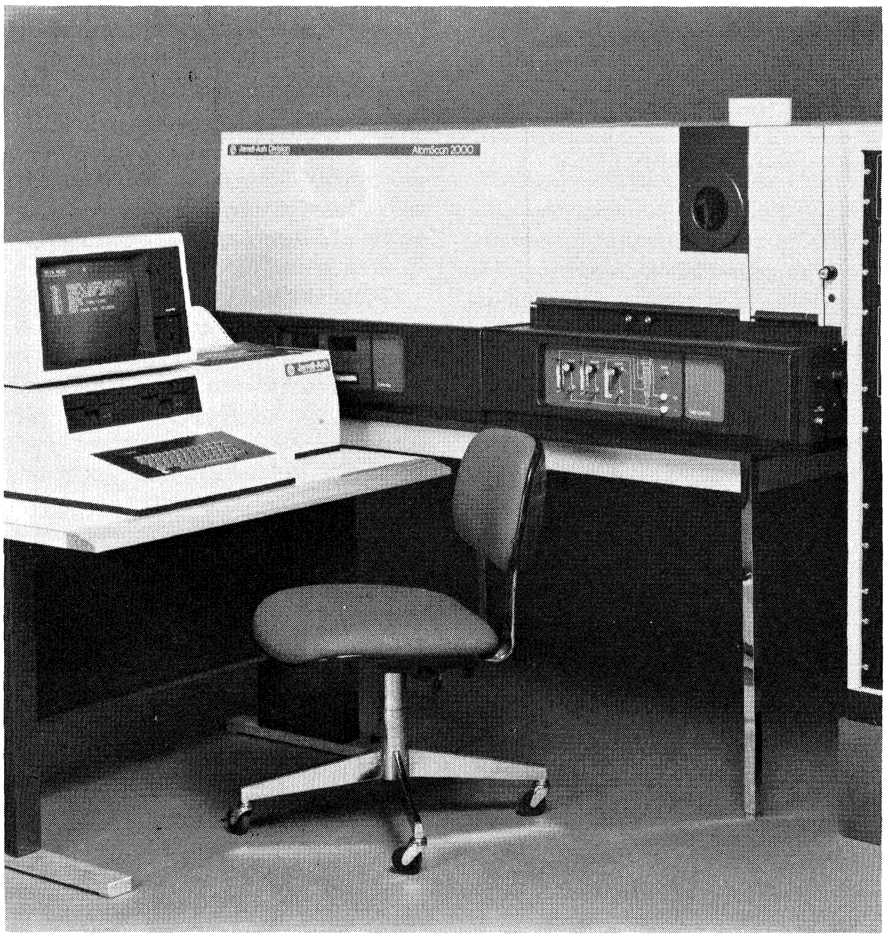

*The AtomScan 2000—an ICAP spectrometer for methods development and single- or multi-element analyses. (Jarrell-Ash Division, Fisher Scientific, Waltham, USA.)*

### Microprocessor-controlled fraction collector

Gilson Medical Electronics' new computer-compatible fraction collector, Model 201, for LC, HPLC and preparative applications can be used as a stand-alone instrument or incorporated in, for example, a Gilson HPLC system.

The Model 201 includes a remote command module with digital display to provide simple touch-switch control of collection mode, rack pattern, wait-time before start of collection, and instrument recycle. Entering of parameters is accomplished through a prompt/respond sequence between the command module and the operator.

The instrument offers drop and time collection modes and several variations of peak collection and variable time-based programming. In its peak modes, the 201 allows selection of any or all peaks above a controllable threshold level, including collection through repeated cycles. Using the variable time program mode, the 201 collects the components detected during the selectable time windows.

*Details from Gilson Medical Electronics Inc., 3000 W. Beltline Highway, Middleton, Wisconsin 53562, USA.*

**Circle No. 19 on Reader Enquiry Card**

### Computer pump

A peristaltic pump, which is suitable for long-term organic perfusion, is the first truly programmable pump with accuracy and repeatability to within 1%. The Compuflow Pump System features digital input and read-out capability and any function can be repeated by panel or by remote switch. It can be programmed to run to pre-set volume or pre-set time. Its safety features include an audible alarm at the end of the operation.

*Information from the Manostat Corporation, 519 Eighth Avenue, New York, NY 10018, USA.*

**Circle No. 20 on Reader Enquiry Card**

### ISCO's autosampler

An autosampler called ISIS can automate sample introduction for AAs, flame photometers and many other analytical instruments. The autosampler can be controlled by an existing computer or by its own, optional, microprocessor sequencer.

ISIS can collect fractions prior to sample transfer and can control accessory equipment, such as a pump, diluter/dispenser, or valve, for simple processing of samples. It holds up to 114 samples and uses 3 to 20 ml containers with or without septum caps. An optional wash station rinses the pipette between every sample.

*Information on ISIS from ISCO Inc., Box 5344, Lincoln, Nebraska 68505, USA.*

**Circle No. 21 on Reader Enquiry Card**

### Multi-step controller from Tecator

The Autostep 1012 is a new time/temperature controller which has been designed for automatic control of wet chemical samples and wet ashing for trace metals for example. The controller features nine temperature levels, which can be programmed for ramp and plateau times of up to 10 h within a temperature range up to 600°C. Times and temperatures are indicated on a digital display. The Autostep 1012 is fully compatible with its manufacturer's line of digestion systems.

*Details from Tecator Inc., 13 Highland Circle, Needham, Massachusetts 02194, USA.*

**Circle No. 22 on Reader Enquiry Card**

### Water purification system—Barnstead Company

NANOpure II is a reagent grade water system which features microprocessor-controlled purity monitoring. The monitoring system indicates when purity has been reached and cartridges should be changed. An LED digital display provides automatic resistivity read-out referenced to 25°C, with temperature display in °C of the water in the system.

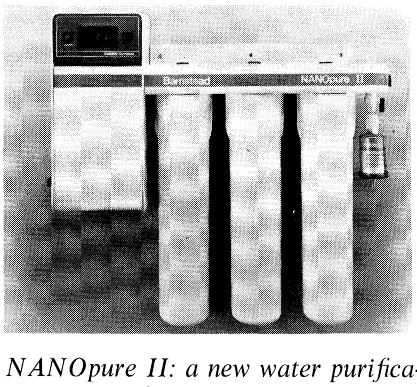

*NANOpure II: a new water purification system featuring microprocessor-controlled monitoring. (Barnstead Company, Boston, USA.)*

Each resistivity reading is checked against two internal standards to ensure proper meter calibration. The NANOpure II also has a pre-treatment cartridge designed to remove colloids, organics and most bacteria. The new cartridge is placed in the first position of three canister systems followed by two mixed-bed ion-exchange cartridges and an autoclavable 0·2 submicron filter. The system produces up to 3 l/min. of 10 to 18 megohm water (depending on the feedwater), and is less expensive to operate than competing systems. It can be used on pre-treated water or high-quality tap-water and is available in a three or four canister configuration. All units meet or exceed NCCLS, CAP and ASTM specifications for Type I Reagent Grade Water.

*Further information from the Barnstead Company, Division of Sybron Corporation, 225 Rivermoor Street, Boston, Massachusetts 02132, USA.*

**Circle No. 23 on Reader Enquiry Card**

### Automatic multi-gas analyser

An industrial mass spectrometer, called the MM8-80, which performs fast continuous and completely automatic analyses of process gases, has been launched by VG Gas Analysis Ltd. The MM8-80 can simultaneously monitor 60 gases from 24 different gas streams and thus eliminates the need to use several analytical techniques to ensure complete monitoring of process plants. The instrument can be used for inlet and outlet gases; the selection of gases to be monitored is simple and can be changed at any time by the operator. The machine is controlled either by a dedicated microprocessor or by a microcomputer which will also handle source selection and data output.

The MM8-80 has a high resistance to contamination and drift and, therefore, periods between recalibration are quite long. Repeat accuracies are better than 1% fsd; typical response time is 250 ms.

The mass spectrometer is intended for use in petroleum production, for the monitoring and control of reaction gases in fermentation, protection of catalysts, monitoring of coal gasification, steel plant monitoring, and ethylene oxide and ammonia production.

*Further information from Brian Scott, VG Gas Analysis Ltd, Nat Lane, Winsford, Cheshire CW7 3QH, UK. Tel.: 06065 52021.*

**Circle No. 24 on Reader Enquiry Card**

### Personal gas and vapour monitor

A personal monitor for gases and vapours, developed at Birmingham University, is available from Dutom Meditech. The monitor is a stainless-steel tube with a unique serial number which is packed to chromatographic standards with absorbents appropriate to the contaminants to be monitored. The instrument is connected to a personal sampling pump which draws air through it at a preset flow rate. Air-borne contaminants are then adsorbed onto the packing material. The monitor is worn clipped to clothing and it can sample for up to 10 h to calculate a time-weighted average exposure. The unit is sealed after use and sent to a laboratory for analysis—collected contaminants are thermally desorbed and analysed using gas chromatography.

*Further information from Dutom Meditech Ltd, 10 Gordon Street, Luton, Bedfordshire LU1 2QP, UK. Tel.: 0582 412354.*

**Circle No. 25 on Reader Enquiry Card**

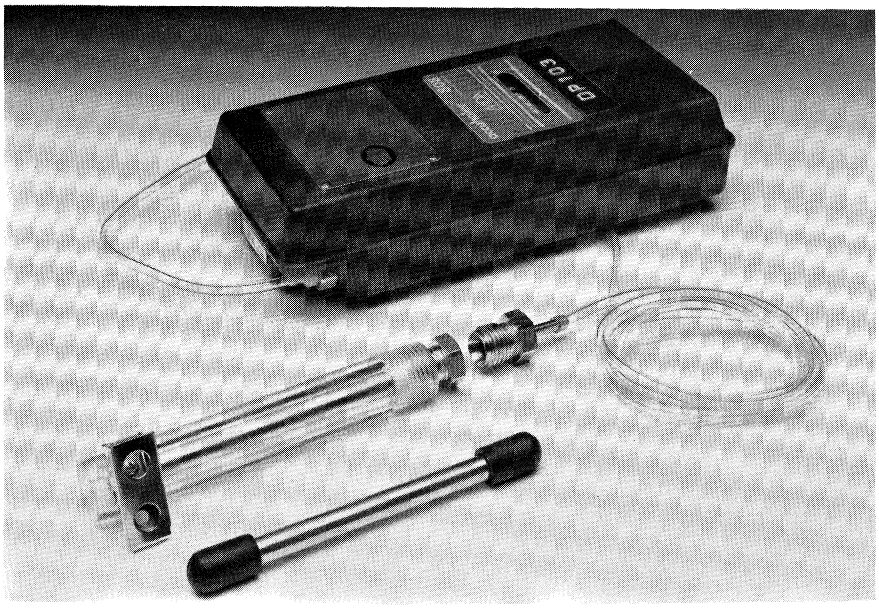

*Dutom Meditech Ltd's Adsorba—a reusable personal monitor for gases and vapours.*

### Compact digital frequency meter

A frequency counter which runs off the mains or a battery has been announced by Simwood Ltd. The new counter, Model MC-841, features gate times from 10 ms to 1 s and has an operational frequency range from 10 Hz to 50 Hz. Model MC-841 can handle square wave inputs between 6 mV and 20 V amplitude.

*Further information from Simwood Ltd, Garretts Hall, Shalford Green, Essex, UK. Tel.: 0371 820006.*

**Circle No. 26 on Reader Enquiry Card**

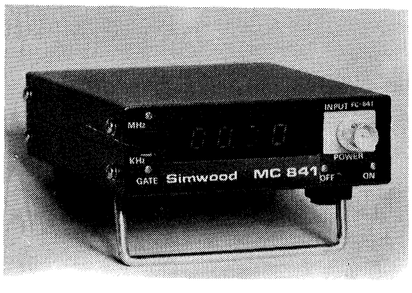

*Model MC-841 frequency counter. (Simwood Ltd, Shalford Green, UK.)*

### The ICP/5500—an inductively coupled plasma system

Perkin-Elmer has announced a new inductively coupled plasma system—the ICP/5500—for analysing trace metals in both low and high concentrations. The system automatically determines up to 80 elements in a sample and up to 15 elements/min. under optimum conditions. The instrument can handle any acid used in the laboratory, as well as high dissolved solids. The newly-designed cross flow nebuliser requires no adjustment and the inert plastic-mixing chamber prevents contamination of samples. The plasma torch is demountable for low running costs and easy maintenance. The ICP/5500 also offers a choice of injectors: quartz for routine applications and high purity alumina for analysing high-concentration hydrofluoric acid solutions. The ICP/5500 is controlled by Perkin-Elmer's Model 3600 Data Station and fully supported by an interactive software package. A new 'Develop' program incorporating high-resolution graphics is used for complete methods development, including wavelength calibrations and background correction parameters.

*Further information from Perkin-Elmer Ltd, Post Office Lane, Beaconsfield, Buckinghamshire HP9 1QA, UK. Tel.: 049 46 6161.*

**Circle No. 27 on Reader Enquiry Card**

### 'Liquid chromatography applications index'

A new bibliography of the extensive range of applications literature on liquid chromatography available from Perkin-Elmer has recently been published. Applications notes are listed under various categories, such as Environment, Pharmaceuticals and Foods, so that references are very easy to find. Copies can be obtained from Martin Perry at Perkin-Elmer Ltd.

**Circle No. 28 on Reader Enquiry Card**

### New ratio-recording infra-red spectrophotometers

Perkin-Elmer have announced a new range of low-cost ratio-recording infra-red spectrophotometers—the 1400 series. Ratio recording guarantees high ordinate accuracy and repeatability, as well as sensitivity to small changes in sample absorption. Full response and accuracy are maintained at low transmission values and the absence of the conventional gain control means that response is always optimized. There are two models in the series: the Model 1420 which covers the range 4000 to 600 $cm^{-1}$, and the Model 1430 which scans from 4000 to 200 $cm^{-1}$. Both instruments have integrated scan controls to prevent selection of meaningless scan conditions and ordinate scale expansion from X1 to X100. The series includes built-in service diagnostics and a purgeable optical path to minimize the presence of water vapour. The models are compatible with the Perkin-Elmer data station, which gives access to a wide range of infra-red applications software including PECDS 11, SEARCH and QUANT.

*For further information contact Perkin-Elmer Ltd.*

**Circle No. 29 on Reader Enquiry Card**

### Portable gas analysers

Columbia Scientific Industries' series 2000/2200 range of portable gas analysers are being distributed in the UK by Techmation. The analysers are simple to use; each has an adjustable, audible alarm to warn of a build up of ozone (1 ppb to 1 ppm) or nitrogen oxides (10 ppb to 50 ppm). Accuracy is better than 2% and response time is under 30s. The models are light and allow up to 10 h of continuous operation from internal batteries.

*Details from Techmation Ltd, 58 Edgware Way, Edgware, Middlesex HA8 8JP, UK. Tel.: 01 958 3111. In the USA contact Columbia Scientific Industries Corporation, PO Box 9908, Austin, Texas 78766, USA.*

**Circle No. 30 on Reader Enquiry Card**

### High voltage power-supply

The HIGHPAC high-voltage power-supply made by Oltronix Labor AG of Biel Bozingen in Switzerland is being distributed in the UK by Wessex Electronics Ltd. The HIGHPAC is intended for bench use or rack mounting and will deliver 0–2250V at 20 mA and up to 2500V with a load current of 10 mA; it is particularly suitable for supplying photomultiplier tubes. The output voltage is continuously variable by means of a 10-turn precision potentiometer, which has digital read-out. Load regulation is 100 mV and line regulation 50 mV; the PARD is less than 2 mV rms. There is a front panel adjustable current limit and either the positive or negative output terminal may be grounded. The 19-in. half-rack case is only 44 mm high.

*Further information from Wessex Electronics Ltd, 114–116 North Street, Downend, Bristol BS16 5SE, UK. Tel.: 0272 571404.*

**Circle No. 31 on Reader Enquiry Card**

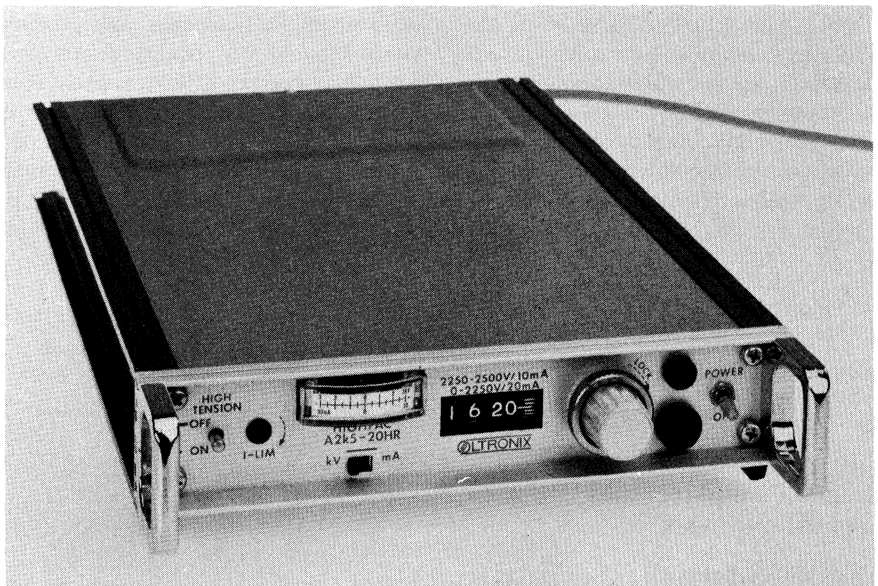

*The HIGHPAC high-voltage power-supply. (Oltronix Labor AG, Switzerland; distributed in the UK by Wessex Electronics Ltd.)*

### Low-cost fridge gas monitor

A continuously monitoring refrigerant leak detector which runs off the mains has been produced by Autoclimate of Birmingham. The 'Gaztell' which is about the size of a telephone, samples the atmosphere for 2·5 s every 2 min. round the clock. Dual flashing lights and an audible alarm operate when the gas concentration reached 1000 ppm—the safety level recommended by the UK's Health and Safety Executive. Autoclimate say that their instrument is so sensitive that it will detect a leakage before refrigeration capacity is affected, preventing danger to food stocks, manufacturing processes, the environment and the plant's workforce.

Gases detected include R11, R12, R14, R22, R113, R502 and other halogens. If the Gaztell master sensing unit is located in a normally unoccupied area it can be linked to a remote alarm, also operated from mains electricity, with identical visual and audible warnings.

The company anticipates demand for the system from the food processing industries, cold stores, supermarkets, and factories where refrigeration is employed in the production cycle; as well as from places with large-scale air-conditioning plants such as offices, airports and computer centres.

*Further information from Autoclimate Ltd, 80 Hatchett Street, Birmingham B19 3NB, UK. Tel.: 021 359 6447.*

**Circle No. 32 on Reader Enquiry Card**

### Update on the CDP1 chromatography computer integrator

Increased calculation power is now available for the low-cost Pye Unicam CDP1 computing integrator, which can

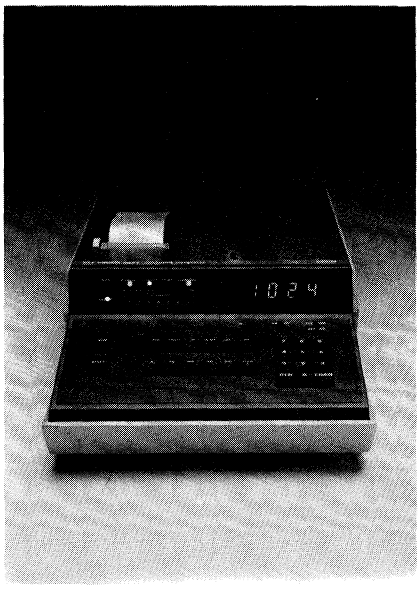

*Pye Unicam's CDP1 computing integrator for gas and liquid chromatography systems, which now has increased calculation power.*

complement many gas and liquid chromatography systems. The new option adds two further advanced calculation possibilities: internal and external standardization. In addition, it doubles the number of possible timed events and allows results to be recalculated using new parameter data. The option is available for new and existing CDP1 integrators; it is economically priced and can be fitted by customers in a few minutes. The CDP1 is a single-channel integrator, which is capable of producing rapid chromatographic data. It is already known for high accuracy, ease of use and flexibility from its choice of peak detection methods and calculations.

*More information on the new option from Pye Unicam Ltd, York Street, Cambridge CB1 2PX, UK. Tel.: 0223 358866.*

**Circle No. 33 on Reader Enquiry Card**

### Eppendorf clinical chemistry analysers

The Eppendorf ACP 5040 is a single-channel, discrete clinical biochemical analyser for the determination of enzymes, substrates, immunoglobulins and EMIT; it is being sold in the UK by Baird & Tatlock Ltd. The instrument has maximum throughput of 300 enzymes/h, and is fully programmable for up to 16 different methods. The ACP 5040 uses small reagent sample volumes, and incorporates an automatic laundry system which washes all the reaction cuvettes, so cost per test is kept to a minimum.

Baird & Tatlock are also marketing Eppendorf's PCP 6121, a semi-automated photometer, which was designed specifically for small laboratories and for use as a back-up instrument. The PCP 6121 is also microprocessor controlled and can store parameters for up to 12 kinetic and end-point methods via a universal keyboard. Like the ACP 5040, the PCP 6121 utilizes a mercury lamp, spectral line principle in its photometeric system. Thus high-intensity spectral lines with an accuracy of $\pm 0{\cdot}1$ nM for each selected wavelength can be achieved.

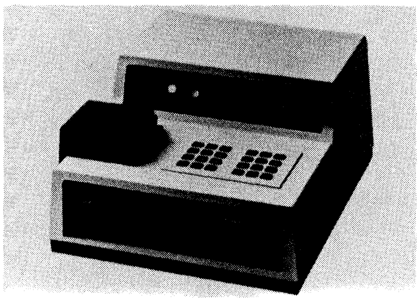

*The PCP 6121 semi-automated photometer. (Baird & Tatlock Ltd.).*

*Further information from Baird & Tatlock (London) Ltd, PO Box 1, Romford RM1 1HA, Essex, UK. Tel.: 01 590 7700.*

**Circle No. 34 on Reader Enquiry Card**

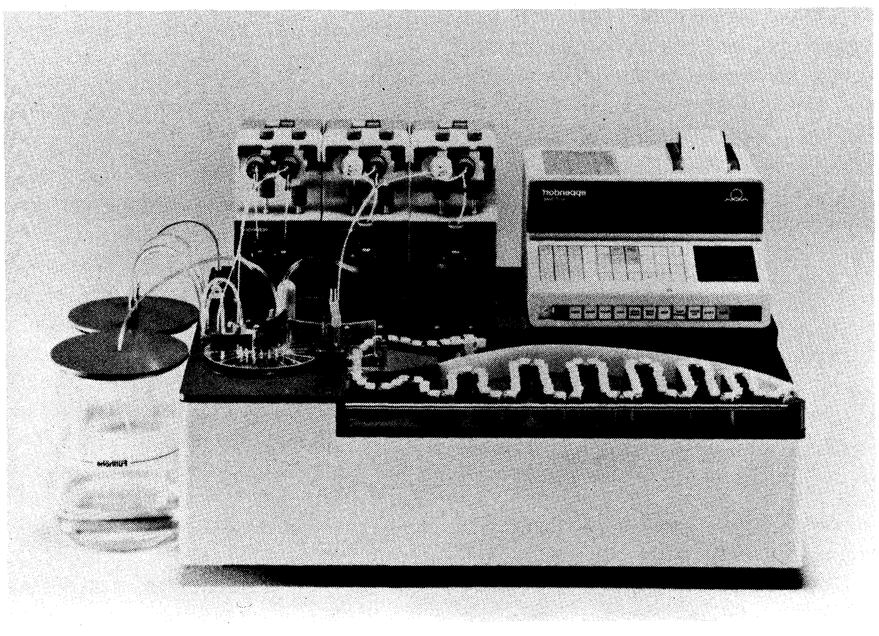

*Eppendorf's ACP 5040 single-channel, discrete clinical biochemical analyser. (Baird & Tatlock [London] Ltd are distributing it in the UK.*

### 'Computer Enhanced Spectroscopy'

Scientists in spectroscopic laboratories all over the world are putting a large amount of effort into spectrometer/computer interfacing and computer programming. There is a real need for a communication medium so that their efforts are cumulative rather than repetitive. It is to provide this vehicle that the journal *Computer Enhanced Spectroscopy* has been launched; it is a journal for the practising laboratory scientist interested in novel work in which the performance of a spectrometer or a chromatograph/spectrometer combination is enhanced with a computer, either for the control of its operation, or for the manipulation of the output data. Contents will be centred on minicomputers and microcomputers, but papers involving more sophisticated computers will be published—especially where the object is to interrelate the output of a number of instruments or to involve databases and spectral collections, or where there are implications for the smaller installation and for general use. In addition to technical papers, the editors propose to publish review articles, short communications and correspondence. *Computer Enhanced Spectroscopy* will have no page charges and 50 free offprints of published material will be supplied to authors. A strict refereeing system will be applied to all submitted material.

Authors wishing to submit a paper should contact the appropriate editor:

*USA*
Dr George Levy, Department of Chemistry, Syracuse University, Bowne Hall, New York 13210.

*Japan*
Professor S. Sasaki, Toyahashi Institute of Technology, School of Materials Science, Tempaku-cho, Toyohashi 440.

*All other countries*
Dr H. A. Willis, The Editorial Office, *Computer Enhanced Spectroscopy*, Heyden & Son Ltd, Spectrum House, Hillview Gardens, London NW4 2JQ.

The journal will appear six times a year. Institutional prices are £50·00, $110·00, DM 230·00; and personal rates are £27·00, $60·00, DM 125·00.

**Circle No. 35 on Reader Enquiry Card**

### Atomic absorption software package

A general-purpose atomic absorption spectrometer software package for Commodore computers is now available from Anaspec. The package has been written with the inexperienced computer user in mind and has clear instructions for its use. It allows several modes of operation, including construction of a calibration curve and calculation of results with reference to this curve. Data-handling facilities allow either direct output to a printer, output to a printer with simultaneous disc storage or output directly to disc for subsequent reuse. Anaspec will soon have other packages on the market—for general chromotography and spectroscopy applications.

*Further information from Anaspec Ltd, Pearl House, Bartholomew Street, Newbury, Berkshire, UK. Tel.: 0635 44329.*

**Circle No. 36 on Reader Enquiry Ca-d**

### The Parallel in Britain

The first Monitor Parallel System has been installed in the Department of Clinical Chemistry at Charing Cross Hospital, London. The Parallel, which is based on American Monitor's KDA analyser, is now in production in Belfast. The system at Charing Cross is one of three which will be evaluated for the ECCLS (the other instruments are to be installed in Lille and in Hannover) and is also being evaluated for the UK's DHSS. The manufacturer intends to have a network of service engineers in the UK backing up the instruments and also to have a number of 'chemistry specialists' around the country on call for advice.

*More information on the Parallel from Mike Clegg, Monitor International, Storrington, Pulborough, West Sussex RH20 3DW, UK. Tel.: 09066 5303.*

**Circle No. 37 on Reader Enquiry Card**

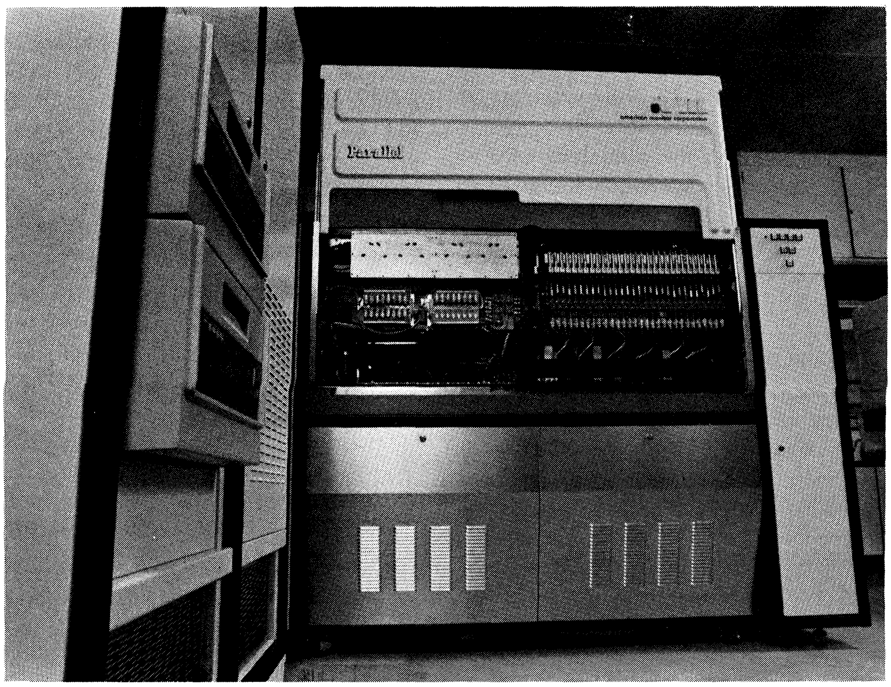

*The Monitor Parallel System installed. The data management towers are in the foreground and the main chemistry console has its front raised to show the sampling and dispensing panels. (Monitor International, Storrington, UK.)*

### Temperature transmitter

Nulectrohms are marketing a programmable temperature transmitter, which is suitable for a wide range of thermocouples. A variety of inputs and outputs can be pre-set by means of small DIP switches and the whole assembly is contained in a plastic case for either DIN rail or surface mounting. The inputs can be mV from any type of thermocouple ranging from copper/constantan to platinum/rhodium, and a wide range of temperature spans can also be pre-set. Offset zeros are possible as the zero can be adjusted from $-50$ to $+500\%$ of selected span by means of a multi-turn potentiometer on the front panel, whilst a further variation of $\pm 20\%$ of the selected span is also available. The output options are as varied as the inputs and can be 0–20, 4–20, 0–50 or 10–50 mA, or 0–5, 1–5, 0–10 or 2–10 V, all of these can be selected with the DIP switches. The unit will accept supply voltages from 110, 220, 240 V a.c., or 24 V d.c., and the input/output power transformer is isolated to 1000 V d.c.; the transmitter is both RF1 and EM1 protected.

*Nulectrohms sell a number of temperature-sensing instruments: thermocouples, resistance thermometers, panel meters and calibration equipment. For details on the new transmitter and their other products contact Nulectrohms Ltd, Meppershall, Shefford, Bedfordshire SG17 5LX, UK. Tel.: 0462 813000.*

**Circle No. 38 on Reader Enquiry Card**

### 'The AutoAnalyst'

March 82's issue of Technicon's house magazine *The AutoAnalyst* includes an article on their RA 1000 system discussing its role in the emergency laboratory and how it fits into small, medium and large labs. Also an interesting announcement of the installation of a second-generation SMAC at the Royal Infirmary of Edinburgh.

The April issue publishes a summary of a paper by D. M. Reardon reporting the results of a six-month evaluation of Technicon's H 6000 haematology system.

*Copies of 'The AutoAnalyst' are available from Technicon Instruments Company Ltd, Evans House, Hamilton Close, Basingstoke, Hampshire, UK.*

**Circle No. 39 on Reader Enquiry Card**

